# PTSD course and predictors in a 15 year longitudinal cohort following suspected serious injury

**DOI:** 10.1038/s44184-025-00153-7

**Published:** 2025-08-07

**Authors:** Jeanet F. Karchoud, Chris M. Hoeboer, Irina Karaban, Joanne Mouthaan, Marit Sijbrandij, Miranda Olff, Rens van de Schoot, Mirjam van Zuiden

**Affiliations:** 1 Amsterdam UMC, University of Amsterdam, Psychiatry, Amsterdam Public Health, Amsterdam, The Netherlands; 2 Leiden University, Department of Clinical Psychology, Leiden, The Netherlands; 3 Department of Clinical, Neuro- and Developmental Psychology, WHO Collaborating Center for Research and Dissemination of Psychological Interventions, Amsterdam Public Health Research Institute, Vrije Universiteit Amsterdam, Amsterdam, The Netherlands; 4 ARQ National Psychotrauma Centre, Diemen, The Netherlands; 5 Utrecht University, Department of Methods and Statistics, Utrecht, The Netherlands; 6 Utrecht University, Department of Clinical Psychology, Utrecht, The Netherlands

**Keywords:** Psychology, Risk factors

## Abstract

Investigating long-term posttraumatic stress disorder (PTSD) course and its predictors may guide prevention and early intervention strategies following trauma exposure, potentially reducing the long-lasting impact of trauma. *N* = 155 emergency-admitted adults with (suspected) serious injury were repeatedly assessed until one-year post-trauma and completed a 12–15 year follow-up including a clinical PTSD interview. Adverse one-year PTSD trajectories; more exposure to additional potentially traumatic events and recent life stressors; and early post-trauma predictors (younger age, greater perceived impact of prior potentially traumatic events, higher heart rate) were significantly associated with higher PTSD symptom severity 12–15 years post-trauma. This study showed high consistency between one-year PTSD and its early post-trauma predictors with long-term PTSD outcomes. Early post-trauma predictors had predictive value up to 12–15 years. This suggests that early risk identification of one-year PTSD and subsequent effective early interventions also hold long-term beneficial effects for PTSD outcome.

## Introduction

Exposure to traumatic events typically causes initial psychological distress that subsides within a few weeks post-trauma^1^. However, for a considerable subset of those exposed, trauma exposure results in posttraumatic stress disorder (PTSD), with an average conditional risk estimated at 4%^1,2^. PTSD symptoms include intrusions, avoidance, negative mood and cognitions, and alterations in arousal and reactivity^1^. PTSD is linked to negative health outcomes and functional impairments, including increased comorbidity and mortality, and reduced (health-related) quality of life^3–6^. Retrospective epidemiological data from the WHO Mental Health Surveys show that the mean duration of PTSD symptoms is six years, with considerable differences in mean duration between trauma types (i.e., higher for war-related experienced, lower for accidents)^7^. This amounts to extensive economic costs and societal burden, besides the individual impact^8^. However, to date, there is only limited prospective longitudinal research addressing long-term PTSD outcomes more than a decade beyond traumatic events.

These prospective longitudinal studies on long-term PTSD symptoms (>10 years) were predominantly carried out among uniformed services staff (e.g., veterans)^9^ and people affected by collective/mass trauma (e.g., tsunami)^10^. In the first long-term longitudinal study following civilians experiencing individual trauma beyond a decade, we recently observed that nearly 5% of adults had long-term PTSD related to previous exposure to a potentially traumatic event resulting in (suspected) serious injury of 12–15 years ago^11^. Higher levels of PTSD symptoms were associated with a broad range of adverse psychological and functional outcomes (e.g., more depressive symptoms, lower quality of life), further underscoring the potential long-lasting impact of individual civilian trauma.

A growing number of longitudinal studies following individual civilian trauma exposure are investigating PTSD symptom trajectories, with the endpoint assessment typically at one-year post-trauma and maximally at six years post-trauma^12,13^. These trajectory-oriented approaches are used to classify individuals into distinct patterns of PTSD symptom severity over time, thus taking the heterogeneous course of PTSD into account. A meta-analytical study identified four commonly observed PTSD symptom trajectories across these studies: resilience (i.e., continuous minimal symptoms), recovery (i.e., high initial symptoms but remitting), chronic (i.e., stable high symptoms) and delayed onset (i.e., increasing symptoms)^13^. It has not been investigated yet how these one-year PTSD trajectories relate to long-term PTSD outcome.

Of note, during the intermittent time period between one-year and long-term follow-up measurements, exposure to additional potentially traumatic events or life stressors may disrupt initially seemingly adaptive PTSD courses (resulting in delayed onset PTSD) or exacerbate already adverse ones^14,15^. Currently, the impact of such additional exposure on the PTSD course has been studied up to two years following individual civilian trauma, compared to 50 years in uniformed services samples^14,15^. However, prevalence rates for delayed onset following trauma in uniformed services are usually higher than following civilian trauma^14^, and particularly those in high-risk occupations are thought to be more often exposed to additional potentially traumatic events and life stressors^16^. Therefore, it remains to be confirmed whether exposure to additional trauma and life stressors is also relevant for long-term PTSD following individual civilian trauma, and if these could explain potential discrepancies between one-year trajectories and long-term PTSD outcome herein.

To date, many prospective and longitudinal studies have identified early post-trauma factors associated with risk versus resilience to adverse one-year PTSD outcome and course (see e.g., umbrella review^17^; and a recent systematic review after traumatic injury^18^). A prospective longitudinal study in a subset of the same TraumaTIPS cohort as investigated in the current study derived the 15 most important contributors to an accurate machine learning-based prognostic risk model for PTSD diagnosis at one-year post-trauma, out of 51 early post-trauma demographic, trauma-related and biomedical variables included in the algorithm given their previously observed univariate predictive value for subsequent PTSD outcome^19^. However, it remains unknown whether these early post-trauma predictors, consisting of demographics (i.e., age); index trauma characteristics (i.e., time of ED admittance; perceived life threat; perceived post-traumatic amnesia); previous trauma characteristics (i.e., amount and perceived impact of prior traumatic events); acute post-trauma physiological measures (i.e., heart rate; systolic blood pressure); acute post-trauma endocrine measures (i.e., cortisol; DHEAS; Thyroid stimulating hormone; Free thyroxine levels at ED admittance); and early post-trauma pharmacotherapy administration (i.e., non-opioid and opioid anesthetics; analgesics), are also associated with long-term PTSD outcome.

The goal of the current prospective longitudinal study was therefore to investigate whether one-year PTSD courses and their previously observed associated predictors were related to long-term PTSD outcome at 12–15 year post-trauma in Dutch adults following (suspected) serious injury. Specifically, we examined whether previously observed one-year PTSD symptom trajectories within the same cohort^20^ were associated with long-term PTSD symptom severity related to the index traumatic event of 12–15 years ago. Additionally, we examined whether this long-term PTSD symptom severity was associated with exposure to additional potentially traumatic events following the index trauma and recent life stressors. Furthermore, we investigated whether the 15 most important early post-trauma predictors of PTSD at one-year post-trauma identified within a subset of the same TraumaTIPS cohort^19^ were also associated with long-term PTSD symptom severity.

## Methods

### Participants and study design

The current study included *N* = 155 trauma-exposed adults (37% women, mean age at follow-up = 54 years, SD = 12.59) with (suspected) serious injury that required transportation by ambulance or helicopter for specialized acute medical evaluation and care to a level-1 emergency department (ED) in Amsterdam, the Netherlands (to former hospitals Academic Medical Center and VU University Medical Center, currently merged into Amsterdam University Medical Center), between 2005 and 2008. Participants were followed up to one-year post-trauma in the prospective TraumaTIPS cohort (‘The Incidence, Prediction and Prevention of Post-trauma Psychopathology Study’)^21^. Participants completed a long-term follow-up assessment at 12–15 years (*M* = 14.30, SD = 1.00) post-trauma. This index traumatic event primarily involved traffic accidents (59.4%), followed by work-related accidents, fall from height, and physical violence (see Supplementary file A for all sample characteristics). Within the current study, we only included those participants who completed two or more clinical PTSD assessments up to one year post-trauma, allowing the estimation of their one-year PTSD symptom trajectories^22^; and who completed the clinical PTSD assessment at the 12–15 years follow-up. Inclusion criteria of the TraumaTIPS cohort were: age 18 years or older; proficiency in Dutch; exposure toa traumatic event according to DSM-IV PTSD A1 criterion. Exclusion criteria were: current severe psychiatric symptoms; moderate-severe traumatic brain injury (i.e., Glasgow Coma Score <13); permanent residency outside the Netherlands. Participants were additionally excluded from the long-term follow-up assessment in case of suspected severe neurological conditions impairing cognition. The TraumaTIPS cohort study was approved by the Medical Ethics Review Committee of the Academic Medical Center and VU University Medical Center hospitals. The larger TraumaTIPS study included a randomized controlled trial (RCT; ISRCTN: 57754429) which evaluated the effectiveness of a brief self-guided internet-based cognitive behavioral intervention for PTSD^21^. Fitting with previous findings of no significant effects of the TraumaTIPS intervention on PTSD symptoms, a non-parametric Kruskall Wallis test showed no statistically significant differences between participants of our current follow-up study who received early intervention (*n* = 33.5%) and those who did not (*n* = 66.5%) in their PTSD symptom severity at 12–15 years post-trauma (*p* = 0.091). The long-term follow-up assessment was exempted from formal ethical review by the Medical Ethical Review Committee of the Amsterdam University Medical Center, location Academic Medical Center (W20_035#20.063).

### Procedures

Upon admission to the ED, hospital staff routinely collected biomedical information (i.e., index trauma characteristics, acute post-trauma physiological and endocrine measures, and early post-trauma pharmacotherapy administration). Blood samples were drawn at ED admittance for stress hormone assessment. Physiological vital measures were concurrently conducted at ED admittance. Potential participants for the TraumaTIPS cohort were identified by screening patient records regarding the inclusion and exclusion criteria. Further eligibility screening was performed in the hospital or via telephone within 72 hours post-trauma. Subsequently, the baseline assessment (T0; <32 days) was preferably scheduled at approximately one week post-trauma, with a maximum of 32 days post-trauma, in which participants gave written and oral informed consent. Moreover, additional screening was performed to exclude participants with current severe psychiatric symptoms using the mini international neuropsychiatric interview (MINI) (MINI; Plus version 5.0)^22,23^. After informed consent, blood samples were analyzed (i.e., cortisol, DHEAS, thyroid-stimulating hormone; free thyroxine), and the following information was obtained from hospital registries: physiological vital measures at ED admittance (i.e., heart rate; systolic blood pressure), index trauma characteristic (i.e., time at ED admittance) and pharmacotherapy administration within 48 hours post-trauma (i.e., non-opioid anesthetics; non-opiate analgesics; opiate anesthetics and analgesics). Participants also completed self-report questionnaires at baseline (T0), including demographics (i.e., age at ED admittance) and additional characteristics related to index- and previous trauma (i.e., self-reported perceived life threat; self-reported perceived post-traumatic amnesia; number of prior potentially traumatic events; perceived impact of prior potentially traumatic events). Follow-up assessment including the Clinician-Administered PTSD scale for DSM-IV (CAPS-IV) (i.e., assessing PTSD symptom severity) were scheduled up to one year post-trauma, preferably at 1 month (T1), 3 months (T2), 6 months (T3), 12 months (T4) post-trauma. Given that assessments frequently occurred later than preferred due to practical issues related to participants’ severe injuries, we redefined the timeframes of the follow-up assessments to the closest appropriate days post-trauma (T1: <60 days, T2: 61–136 days, T3: 137–273 days, T4: >273 days post-trauma)^20^. See^21^ for more details regarding these procedures.

Participants of the TraumaTIPS cohort who provided permission to be contacted again for follow-up assessments as part of the informed consent and had not formally withdrawn their informed consent, were invited for the long-term follow-up assessment. They were approached via telephone and e-mail using available contact information. Upon successfully verifying their identity (based on personal details and index trauma) and eligibility of study participation via telephone, participants received study information via e-mail including a secure link to an informed consent form (Castor electronic Data Capture (EDC), 2019). After providing informed consent, participants completed the follow-up assessment 12–15 years post-trauma (T5). The clinical interview Clinician-Administered PTSD scale for DSM-5 (CAPS-5) related to the index trauma of 12–15 years ago was administered via video conferencing, including the Life Events Checklist for DSM-5 (LEC-5) for exposure to additional potentially traumatic events. They received a personal link to several online self-report questionnaires in Castor EDC, including recent life stressors exposure, and sociodemographic outcomes.

There was selective dropout in the follow-up assessment at 12–15 years post-trauma compared to all *N* = 852 participants of the original TraumaTIPS cohort^21^. Participants who completed the follow-up assessment exhibited significantly lower PTSD symptoms and less often met the diagnostic criteria for PTSD at one-year post-trauma; had higher participation rates in the intermediate assessments; and at baseline reported less perceived impact of prior potentially traumatic events, less often a non-Dutch origin, higher education levels, and more often being in a relationship compared to those who did not participate^11^. Out of the *N* = 554 participants of the original *N* = 852 who were previously assigned to a one-year PTSD symptom trajectory^20^, *n* = 399 (72.02%) participants did not complete the follow-up assessment at 12–15 years post-trauma. All participants who were previously assigned to one-year PTSD trajectories did not differ from participants who completed the follow-up assessment in terms of adaptive (i.e., resilient; recovery) vs. adverse (i.e., chronic; delayed) one-year PTSD symptom trajectory assignment (Fisher’s exact test, *p* = .063).

### CAPS-IV

The Dutch version of the CAPS-IV was used to measure PTSD symptom severity over the last month at each follow up assessment up to one-year post-trauma^24,25^. The CAPS-IV includes 17 items corresponding to DSM-IV symptom criteria for PTSD (re-experiencing five items; avoidance seven items; hyperarousal five items), assessing both frequency and intensity of each symptom on a 4-point Likert scale, ranging from 0 ‘absent’’ to 4 ‘extremely.’’ CAPS-IV total scores were calculated by summing frequency and intensity scores for all 17 items (range 0–136). The CAPS-IV has excellent internal consistency^24,25^.

As outcome we used one-year latent PTSD symptom trajectories (i.e., resilient, recovery, chronic, delayed) of CAPS-IV total scores across follow-up that were previously assigned to each participant with at least two CAPS-IV assessments available^20^. These trajectories were calculated using sex-stratified Bayesian Latent Growth Mixture Modeling with informative priors for the growth parameters of the intercept (T1) and linear slope (T2-T4), derived from a systematic review and expert elicitation on latent PTSD trajectories^26^; and priors on the expected size of each PTSD trajectory based on mean prevalence rates of a meta-analytic study^13^.

Due to the time gap between the last and current follow-up assessment spanning more than a decade, with no information available regarding the intermittent period, we refrained from performing novel latent trajectory analyses across the whole follow-up period and instead related the previously derived one-year trajectories to long-term PTSD outcome. Latent trajectory analyses on the original one-year follow-up period were already performed within the current cohort^19,20^.

### CAPS-5

We used the Dutch validated version of the standardized CAPS-5 to measure PTSD diagnosis and PTSD symptom severity over the last month, assessed at the 12–15 years follow-up^27–29^. The CAPS-5 was measured in relation to the index trauma of 12–15 years ago. Participants were specifically instructed to keep in mind the index trauma of 12–15 years ago in answering the questions. The CAPS-5 includes 20 items corresponding to DSM-5 symptom criteria for PTSD, measured on a 4-point Likert scale, ranging from 0 ‘absent’ to 4 ‘extremely’. Participants met the diagnostic criteria for PTSD when scoring 2 or higher on at least one intrusion item and avoidance item, two items on negative alterations in cognitions and mood, and two hyperarousal items; had symptoms for over a month; and reported impairment of functioning^29^. In accordance with the CAPS-5 manual, we only counted symptoms with definite or probable connections as PTSD symptoms attributed to the index trauma of 12–15 years ago. We additionally calculated a CAPS-5 total score by summing the 20 items (range 0–80 with higher scores reflecting higher symptom severity). To ensure interrater reliability, a random selection of 15% of the audiotaped CAPS-5 interviews was re-scored each month by a second independent rater. We observed high internal consistency of the CAPS-5 total score (Cronbach’s *α* = .89), as well as excellent inter-rater agreement on PTSD diagnosis (Cohen’s kappa = 1) and PTSD symptom severity (ICC = 0.80).

### LEC-5 trauma exposure

Exposure to additional potentially traumatic events was assessed with the LEC-5, as part of the CAPS-5 diagnostic interview^27,28,30^. Participants were asked about 16 categories of trauma exposure they directly experienced, witnessed, encountered during work or learned about happening to a close friend or family member after the index trauma of 12–15 years ago. We calculated a total score of trauma exposure by summing the 16 categories across and 4 subcategories (range 0–64)^31^

### Recent life stressors exposure

We measured life stressor exposure over the past 12 months. The checklist for life stressor exposure over the past 12 months was based on a previously published checklist, including the following seven life stressors: inability to pay for food, housing, or basic necessities for three months or longer; health problems of someone close; providing care for someone close; conflict at work or study; losing job or study; legal problems; losing someone close due to health, older age, accident, murder, suicide^32^. We added the following 4 exposures based on a previous stressful life events list^33^: interpersonal conflict with someone close; romantic relationship problems or ending of relationship; losing a pet; moving house. A total score of recent stressors was calculated by summing all categories of the checklist (range 0–11).

### Early post-trauma predictors

Age (in years) at ED admittance was obtained from hospital records^21^.

Time at ED admittance, used as a proxy for the timing of trauma exposure, was obtained from hospital records^21^. For the current analyses, we re-indexed the time at ED admittance item from minutes since midnight to the Zeitgeber time in minutes since sunrise to better reflect the circadian rhythm of blood cortisol levels (highest around sunrise and decreasing over day)^34^. Zeitgeber time was calculated using data from the Royal Netherlands Meteorological Institute (KNMI) regarding the time of sunrise on the day of participants’ ED admittance. Perceived life threat and perceived post-traumatic amnesia related to the index trauma^21^ were assessed at T0, using the binary self-report items (yes/no) ‘whether they thought they would die’’ and ‘whether everything was well remembered.’’

The number of prior traumatic events and perceived impact of these prior potentially traumatic events were measured at T0 using the List of Traumatic Events^35^. Participants were asked how many prior traumatic events they experienced prior to the index traumatic event based on a list of 26 trauma exposure types. A total score was calculated for the number of prior traumatic events (range 0–26). For each type of event experienced, participants indicated their perceived impact of these events on a 5-point Likert scale, varying from no negative impact (0) to very high negative impact (4). A total score was calculated for the perceived impact of prior potentially traumatic events (range depends on number of prior traumatic events with a highest possible range of 0–104).

Heart rate and systolic blood pressure were measured by hospital staff at ED admittance^21^.

Cortisol, DHEAS, thyroid stimulating hormone and free thyroxine were measured from blood samples drawn by hospital staff at ED admittance^36^. Blood samples were stored at −80 °C in 4.5 mL cryovials and analyzed in consecutive batches. To quantify cortisol levels (nmol/L), chemiluminescence assay was utilized using the Immulite 2000 system (Siemens, Breda, the Netherlands). The coefficient of variation between different batches (i.e., inter-assay) was 5.5%, and 8.3% within the same batch (intra-assay). DHEAS levels (nmol/L) were analyzed using Radioimmunoassay (RIA; Siemens), with 4.4% inter-assay and 6.3% intra-assay variation coefficients. Thyroid-stimulating hormone (mE/L) and free thyroxine (pmol/L) were measured with fluorescence immunoassays (Perkin Elmer, Germany). Inter- and intra-assay variation coefficients were all below 10%.

Non-opioid anesthetics; non-opiate analgesics; opiate anesthetics and analgesics administration within 48 hours post-trauma was documented in hospital records^37^. Medication administration within each category was measured in the number of dosages. Non-opioid anesthetics include for example, propofol, etomidate, ketamine, sevoflurane and desflurane. Examples of non-opiate analgesics include for example paracetamol, ibuprofen, naproxen, aspirin and diclofenac. Opiate anesthetics and analgesics include, for example morphine, oxycodone, fentanyl, codeine and hydromorphone.

### Statistical analyses

The statistical analyses plan was pre-registered on OSF^38^. Statistical analyses were performed using IBM SPSS Statistics Version 28.0 and R version 3.6.1.

There was no missing data in the long-term follow-up assessment due to forced entry of questionnaire item responses. The percentage of missing data in early post-trauma predictors ranged from 0 to 66.5%, see Supplementary file B for an overview. We used multiple imputation of missing data as this is a recommended strategy to impute plausible values for (high) missing data in clinical research^39^. Missing early predictor data was imputed using multiple imputation by chained equations (MICE) using the R package “mice,’’ iterated through 20 cycles^40^. In addition to including all variables included in the subsequent analysis, auxiliary variables were selected with a minimum correlation of 0.3 (as recommended in^40^) from *k* = 224 variables assessed in the TraumaTIPS cohort within <62 days post-trauma based on their hypothesized prospective and concurrent predictive value for PTSD symptoms. The default correlation setting of 0.3 resulted in at least multiple (auxiliary) predictor variables per early predictor variable with missing data (see Supplementary file C for predictor matrix for multiple imputation).

Aligning with the expected absence of high PTSD symptoms in the majority of our sample at long-term follow up^13^, CAPS-5 total scores were heavily right skewed. Therefore, we used a non-parametric ANOVA followed by a Benjamini-Hochberg corrected^41^ post-hoc Kruskal Wallis Tests to compare CAPS-5 total scores at 12–15 years post-trauma in participants assigned to the one-year PTSD symptom trajectories (i.e., resilient, recovery, chronic, delayed). This resulted in a Benjamini-Hochberg alpha correction of *α* = .033 to correct the false discovery rate for multiple comparisons.

Following previous recommendations for analyzing right-skewed data^42^, we conducted two Poisson regression analyses to examine associations of CAPS-5 total score with exposure to additional potentially traumatic events and recent life stressors, and with early post-trauma predictors. The Poisson assumption of equidispersion was not met, as the variance of the CAPS-5 was larger than its mean (dispersion = 5.48 i.e., overdispersion). Therefore, we used Bayesian Poisson regression analyses, which is capable in dealing with overdispersion whilst not assuming qualitatively different response categories between the value 0 and the other low values of the dependent variable (which is the case in the commonly used negative binominal regression and zero-inflated Poisson)^43,44^; and well-suited for our relatively modest sample size^45–47^. We used the R package ‘arm’ to conduct Bayesian generalized linear models (i.e., bayesglm) with a Poisson distribution using standard priors^48^. Moreover, we found indications of multicollinearity among the 15 included predictors, as a Pearson correlation matrix (see Supplementary File D) revealed high correlations between nonopioid anesthetics, nonopiate analgesics and opiate (*r* = 0.99; 0.71; 0.70), and between the number of prior traumatic events and perceived impact of prior traumatic events (*r* = 0.77). Therefore, the main analysis was performed without the least important variables of those with high correlations (i.e., nonopiate analgesics; opiate and prior traumatic events) according to the previous study reporting these 15 most important predictors^19^. We conducted a pooled Bayesian Poisson regression on CAPS-5 total scores with the following 12 out of 15 previously identified early post-trauma predictors: age at ED admittance, time at ED admittance, self-reported perceived life threat, perceived post-traumatic amnesia, perceived impact of prior potentially traumatic events, heart rate, systolic blood pressure, cortisol, DHEAS, thyroid-stimulating hormone, free thyroxine, and non-opioid anesthetics dosage.

Furthermore, we conducted a Bayesian Poisson regression on CAPS-5 total scores predicted from LEC-5 exposure to additional potentially traumatic events and recent life stressor exposure. A sensitivity analysis was conducted in participants who were assigned to the one-year resilient PTSD symptom trajectory to see whether the effect holds as a potential explanation for delayed onset PTSD.

## Results

### Long-term PTSD course

**Fig. 1:**
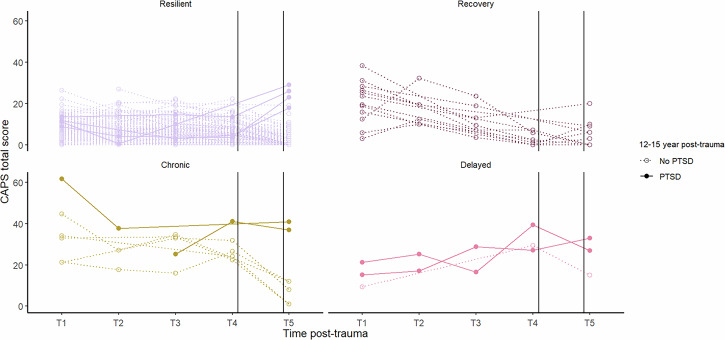
Long-term PTSD Course. The long-term PTSD course is presented for each individual participant. The CAPS-IV total score was used for PTSD symptom severity up to one year post-trauma (Assessments T1-4) and the CAPS-5 was used at 12–15 years post-trauma (Assessment T5). Participants who met the diagnostic criteria for PTSD at 12–15 years post-trauma are represented with bold lines and solid dots, and we used dotted lines and open circles for those who did not meet the diagnostic criteria for PTSD at 12–15 years post-trauma. Participants were categorized into their assigned one-year PTSD symptom trajectory: resilient, recovery, chronic or delayed.

**Fig. 2:**
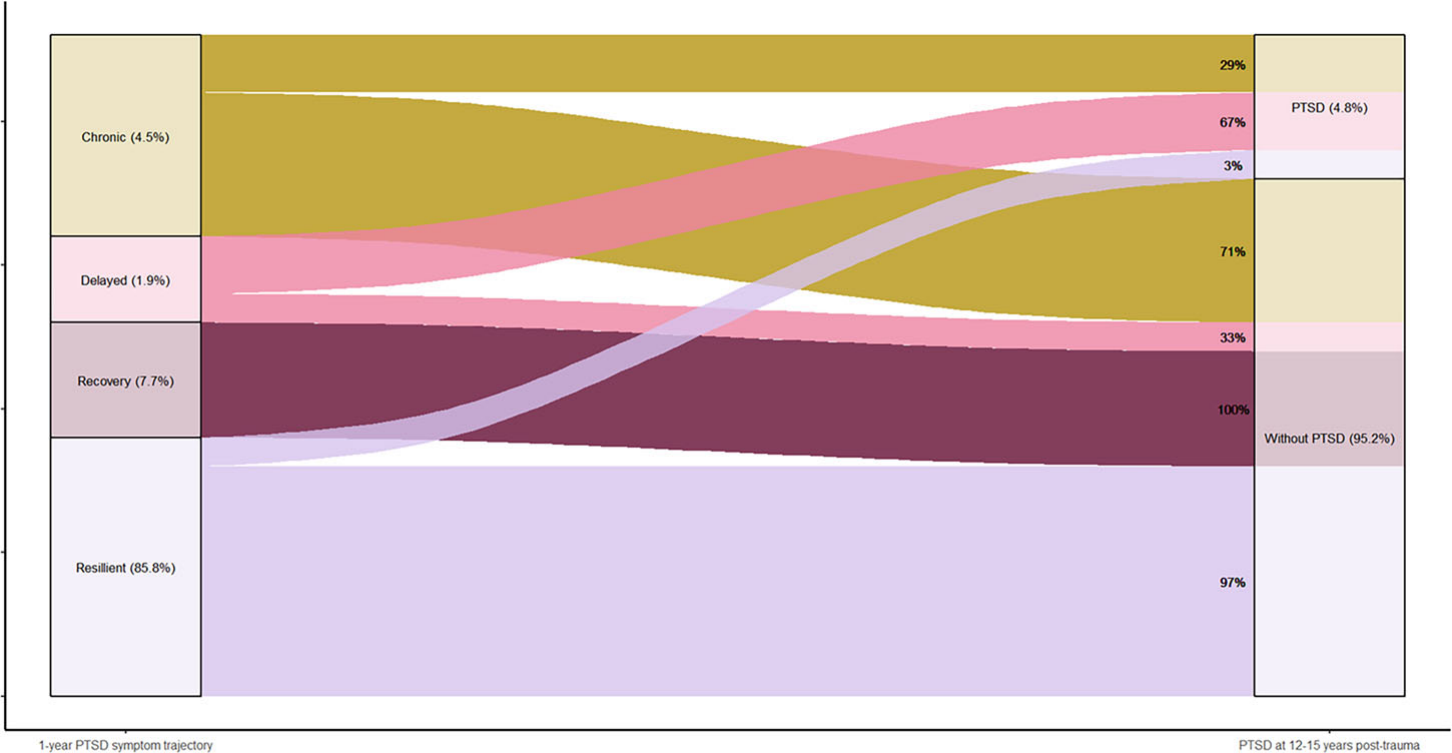
Long-term PTSD outcome. The long-term PTSD outcome at 12–15 years post-trauma is presented for each group of participants assigned to the chronic, delayed, recovery or resilient one-year PTSD symptom trajectory.

See Fig. 1 for the long-term PTSD course for all individual participants showing CAPS total scores at each available assessment, the assigned one-year PTSD symptom trajectory and DSM-5 diagnostic status for PTSD at 12–15 years post-trauma. Of the *n* = 8 participants who met the diagnostic criteria for PTSD at 12–15 years post-trauma, *n* = 4 (50%) were previously assigned to the resilient; *n* = 2 (25%) to the chronic; *n* = 2 (25%) to the delayed onset; and *n* = 0 (0%) to the recovery one-year PTSD symptom trajectories. See Fig. 2 for percentages of participants within each one-year trajectory who did and did not meet the diagnostic criteria for PTSD at 12–15 years post-trauma. Of the participants who were assigned to the one-year resilient trajectory, the large majority of participants (*n* = 129, 97%) did not meet the diagnostic criteria for PTSD at 12–15 years post-trauma. None of the 12 participants who were assigned to the one-year recovery trajectory; two participants (67%) assigned to the one-year delayed trajectory and two participants (29%) assigned to the chronic trajectory met the diagnostic criteria for PTSD at 12–15 years post-trauma.

### One-year PTSD symptom trajectories

**Fig. 3:**
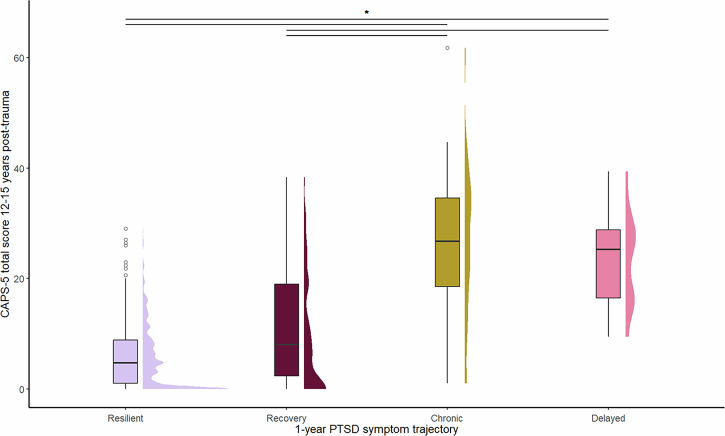
The long-term PTSD symptom severity between one-year PTSD symptom trajectories. The long-term PTSD symptom severity was measured using the CAPS-5 total scores at 12–15 years post-trauma and compared between the groups of participants assigned to the resilient, recovery, chronic or delayed one-year PTSD symptom trajectory. This was presented using boxplots displaying the range, median, 25 and 75th percentile boxes, and distributions of frequencies. The significant post-hoc effects are presented with an asterisk and were based on a Benjamini-Hochberg alpha correction of *α* = 0.033.

CAPS-5 total scores at 12–15 years post-trauma differed significantly between participants within the four distinct one-year PTSD symptom trajectories (Kruskal Wallis Test Statistic = 18.60, *p* < 0.001; see Fig. 3). Benjamini-Hochberg corrected post-hoc comparisons revealed that participants assigned to the resilient trajectory had significantly lower CAPS-5 total scores at 12–15 years post-trauma than those in the chronic (Kruskal Wallis Test Statistic = −52.27, *p* = 0.002) and delayed trajectories (Kruskal Wallis Test Statistic = −75.91, *p* = 0.002). Likewise, participants in the recovery trajectory scored significantly lower on CAPS-5 total scores at 12–15 years post-trauma than those in the chronic (Kruskal Wallis Test Statistic = −44.73, *p* = 0.028) and delayed trajectories (Kruskal Wallis Test Statistic = −68.38, *p* = 0.013). There were no significant differences between participants in the recovery and resilient trajectories (Kruskal Wallis Test Statistic = −7.54, *p* = 0.559), nor between participants in the delayed and chronic trajectories (Kruskal Wallis Test Statistic = −23.64, *p* = 0.423).

### Exposure to potentially traumatic events and recent life stressors

Higher LEC-5 total score trauma exposure since index trauma (posterior estimate = 0.08, SE = 0.01, *p* < 0.001, rate ratio = 1.08), and higher recent life stressors exposure at 12–15 years post-trauma (posterior estimate = 0.23, SE = 0.02, *p* < 0.001, rate ratio = 1.26) were significantly associated with higher CAPS-5 total scores at 12–15 years post-trauma. The sensitivity analysis in participants assigned to the one-year resilient trajectory showed the same effects (see Table 1).

**Table 1:** Overview of Poisson regression analysis of LEC-5 trauma exposure and recent life stressors exposure on CAPS-5 total score at 12–15 years post-trauma, conducted in all participants and a sensitivity analysis in participants assigned to the 1-year resilient PTSD symptom trajectory

	*M (SD)*	Posterior estimate	*SE*	*p*	Rate ratio	95% CI
**In all participants (*n*** = **155)**						
LEC-5 total score trauma exposure	2.94 (2.63)	0.08	0.01	<0.001*	1.08	0.05;0.10
Recent life stressors exposure	1.37 (1.52)	0.23	0.02	<0.001*	1.26	0.19;0.28
**Only in one-year resilient PTSD symptom trajectory (*n*** = **133)**						
LEC-5 total score trauma exposure	2.80 (2.54)	0.07	0.02	<0.001*	1.07	0.03;0.10
Recent life stressors exposure	1.21 (1.40)	0.19	0.03	<0.001*	1.20	0.12;0.24

Note. LEC-5 = Life Events checklist for the DSM-5. All continuous variables are standardized.

* = Significant effect (*p* < .05).

### Early post-trauma predictors

The pooled findings revealed four significant early post-trauma predictors (see Table 2): younger age at ED admittance (pooled posterior estimate = −0.19, SE = 0.05, *p* < 0.001, rate ratio = 0.83), greater perceived impact of prior potentially traumatic events (pooled posterior estimate = 0.29, SE = 0.06, *p* < .001, rate ratio = 1.33), and higher heart rate at ED admittance (pooled posterior estimate = 0.35, SE = 0.09, *p* = .001, rate ratio = 1.42) were significantly associated with higher CAPS-5 total scores at 12–15 years post-trauma.

**Table 2:** Overview of pooled Poisson regression analyses of early post-trauma predictors on CAPS-5 total score at 12–15 years post-trauma

	M (SD)	Pooled posterior estimate	SE	p	Rate ratio	95% CI of pooled posterior estimate
**Demographics**						
Age at ED admittance (in years)	40.82 (12.32)	−0.19	0.05	<0.001*	0.83	−0.29;−0.09
**Index trauma**						
ED admittance in minutes since sunrise	587.25 (362.98)	−0.12	0.08	0.158	0.88	−0.30;0.05
Perceived life threat (yes)	18.1%	0.23	0.14	0.115	1.25	−0.06;0.51
Perceived post-traumatic amnesia (yes)	51.6%	0.14	0.10	0.147	1.15	−0.05;0.33
**Prior trauma**						
Total impact of prior traumatic events	3.47 (3.62)	0.29	0.06	<0.001*	1.33	0.17;0.41
**Physiological measures**						
Heart rate at ED admittance	82.97 (18.21)	0.35	0.09	0.001*	1.42	0.16;0.54
Systolic blood pressure at ED admittance	142.99 (22.08)	−0.09	0.09	0.314	0.91	−0.28;0.09
**Endocrine measures**						
Cortisol (nmol/L) at ED admittance	709.82 (269.29)	−0.04	0.11	0.744	0.96	−0.27;0.20
DHEAS (nmol/L) at ED admittance	4.51 (3.29)	0.13	0.10	0.219	1.14	−0.09;0.34
Thyroid stimulating hormone (mE/L) at ED admittance	2.17 (1.80)	0.09	0.14	0.531	1.10	−0.23;0.41
Free thyroxine (pmol/L) at ED admittance	14.49 (2.61)	0.08	0.11	0.479	1.08	−0.15;0.31
**Pharmacotherapy administration**						
Non-opioid anesthetics administration within 48 h post-trauma (number of dosage)	1.18 (1.20)	0.07	0.09	0.445	1.07	−0.12;0.26

Note. ED = Emergency Department. All continuous variables are standardized. * = Significant effect (*p* < .05).

## Discussion

The goal of the current study was to examine whether one-year PTSD course and its predictors were associated with long-term PTSD outcome (>10 years) in a prospective longitudinal cohort of adults with (suspected) severe injury. We found that one-year PTSD symptom trajectories, exposure to additional potentially traumatic events since the index trauma and recent life stressors were associated with higher PTSD symptom severity related to the index trauma of 12–15 years ago. Out of the early post-trauma predictors, younger age at trauma; greater perceived impact of previous potentially traumatic events; and higher acute post-traumatic heart rate were associated with higher long-term PTSD symptom severity.

Participants following chronic and delayed one-year PTSD symptom trajectories had higher PTSD symptom severity at 12–15 years post-trauma, compared to those following resilient and recovery trajectories. Thus, those with high PTSD symptom severity at one-year post-trauma (i.e., adverse vs. adaptive trajectories) also experienced higher long-term PTSD symptom severity. This implies high consistency in one-year and long-term PTSD outcome. However, descriptively, there was no complete overlap in adverse vs. adaptive individual one-year trajectory assignment and PTSD diagnostic status at 12–15 years. A potential explanation can be found in our results showing that additional exposure to potentially traumatic events after the index trauma and recent life stressors was associated with higher long-term PTSD symptom severity. These effects were replicated in the sensitivity analysis containing only participants following one-year resilient trajectories. This tentatively suggests that these additional exposures are not only associated with the maintenance or worsening of already (pre)clinical PTSD symptom levels over time, but also with the actual long-term delayed onset of PTSD symptoms. This is consistent with cognitive models of PTSD^49^. Exposure to potentially traumatic events after the index trauma and recent life stressors may reinforce excessively negative appraisals of the trauma, impairing adaptive memory processing of the traumatic event. Although we only considered PTSD symptoms with definite or probable index trauma-relatedness, it remains possible that participants reported symptoms due to other traumatic experiences or life stressors. Nevertheless, the observation of this effect following individual civilian trauma adds to the existing literature base on explanatory factors for long-term delayed PTSD (symptom) onset mostly consisting of samples with uniformed services^14,15^.

We found that several previously established early post-trauma predictors of PTSD diagnostic status at one-year post-trauma within the same cohort^19^ were also associated with PTSD symptom severity 12–15 years post-trauma. These findings of prolonged predictive effects of younger age at trauma, greater perceived impact of previous potentially traumatic events, and higher acute post-traumatic heart rate for long-term PTSD symptoms build upon multiple systematic reviews and meta-analyses relating these factors to increased PTSD risk in cross-sectional or longitudinal studies with shorter follow up durations^17,18,50,51^. There were no significant effects of endocrine measures, early post-trauma amnesia and pharmacotherapy administration early post-trauma on PTSD symptom severity 12–15 years post-trauma. This could be partially explained by our current use of a relatively simple multivariate linear regression model instead of the previously used machine learning model containing multivariate non-linear interactions between included predictors^19^. We also need to be cautious in interpretating the findings of the endocrine measures (i.e., cortisol; thyroid stimulating hormone; free thyroxine; DHEAS) given its high percentage of missing data (49.7–66.5%). We encourage future studies to investigate the underlying mechanisms behind these associations by directly examining potential mediation effects of the identified predictors. For instance, higher heart rate could indicate higher perceived severity of injuries. Exploring potential mediating effects could deepen our understand of how early responses to trauma contribute to long-term PTSD outcomes.

Previous literature provides indications for neurocognitive processes that may potentially underlie the observed associations between early post-trauma predictors and long-term PTSD outcome. For example, the observed effect of age may be (partially) explained by developmental changes in adaptive emotion regulation^52,53^. The predictive effect of heart rate as marker for sympathetic activation^51^ for long-term PTSD tentatively align with previous findings^54^. Within the previous one-year follow-up study^19^ prognostic models for PTSD diagnosis and latent PTSD symptom trajectories achieved equally good prognostic accuracy, and 2/3rd of the most relevant predictors within these models overlapped^19^. In future studies, focusing on distinct long-term PTSD courses and their non-overlapping predictors could shed additional light on these etiological mechanisms and provide future therapeutic targets for early interventions for PTSD following trauma.

There are limitations of our study that need to be considered. As there was a considerable time gap between our final and pre-final assessments, it remains unknown whether participants with high one-year and long-term PTSD symptoms continuously experienced consistent PTSD symptoms since the one-year assessment, or whether they may have shown periods of intermittent (partial) symptom remission or worsening; and vice versa for those with low symptoms. Furthermore, as we did not collect information on potential received treatment, the potential impact of these on PTSD trajectories and 12–15 year PTSD symptom severity could not be estimated. Future research should examine the impact of seeking and receiving evidence-based psychological or pharmacological treatment on subsequent symptom course in longitudinal cohort studies. Furthermore, we were not able to explicitly investigate and predict actual changes in PTSD symptom severity between the one-year and 12–15 year assessments. Although research reported a moderate association between the CAPS-IV and CAPS-5^29^, the revised DSM diagnostic criteria and relatedly the applicable CAPS interview version between previous assessments hampers direct comparison. In particular, the inclusion of new DSM-5 symptom cluster negative alterations in mood and cognition as well may have affected measurement consistency.

Our longitudinal study had (selective) drop-out and a modest sample size. Although adaptive vs. adverse one-year trajectories were not associated with dropout in itself, of particular relevance is that participants completing the long-term follow-up had significantly lower PTSD symptoms and lower likelihood of PTSD diagnosis at one-year post-trauma as well as higher participation rates in the intermediate assessments up to one-year post-trauma compared to participants who did not^11^. Also considering that the assigned one-year PTSD trajectories only included participants with at least two completed intermediate follow-up assessments^20^, this suggests an underrepresentation of participants with adverse one-year trajectories within this study. This potentially limits the generalization of our sample to the wider population of adults exposed to (suspected) serious injury, and particularly to higher risk groups of chronic or delayed onset PTSD. It is also plausible that there are differences in the representation of risk factors among underrepresented groups within our sample. For example, individuals with a lower educational background or non-Dutch origin could experience a higher perceived life threat because of communication barriers in understanding medical procedures, or receive less pharmacotherapy because of bias or communication barriers (see systematic review on pain and inadequate pain management in racialized minorities and individuals of lower socioeconomic status^55^). Moreover, the current study did not have enough statistical power to derive or validate a novel prognostic machine learning model for PTSD^19^, resulting in the current investigation of a more limited number of 12 previously identified prognostic variables within the same population, and therefore missing other important previously observed predictors related to PTSD. We also lacked statistical power to employ gender-sensitive analyses in men and women separately. This is particularly relevant as we did not observe significant effects of gender on long-term PTSD prevalence and PTSD symptom severity in our 12–15 year follow-up^11^, while we previously observed significant gender differences in one-year trajectory prevalence and within-trajectories course applied in the current study^20^. Moreover, an increasing number of studies indicated gender differences in the PTSD course and its associated risk factors^56,57^.

Our findings may hold clinical implications for preventive or early post-trauma interventions in PTSD and associated research. Early accurate identification of individuals at risk for developing PTSD is considered to be crucial for effective targeted preventive interventions^58–60^. Although external validation and clinical implementation remains limited, multiple computational studies in recently trauma-exposed civilians demonstrate accurate PTSD risk prognosis for one-year outcomes based on early post-trauma predictors (see meta-analysis^61^). Within our study, we observed that one-year trajectories were strongly related to long-term PTSD outcome, with the large majority of participants showing consistency in whether their trajectory within the first year and 12–15 years later were adaptive or adverse. Although no causal claims can be made from our observational analyses, our findings also suggest that early post-trauma predictors may not only be involved in the development of PTSD symptoms and its one-year course but also more long-term PTSD outcome. This tentatively implies that early risk identification for one-year PTSD and subsequent targeted early interventions towards these individuals at risk, also holds relevance for long-term PTSD outcome and associated adverse psychological and functional outcomes^11^. As the current study was the first in its kind to investigate long-term PTSD course and its early post-trauma predictors, its replication, as well as investigation of the findings’ generalizability within a variety of trauma-exposed populations, are recommended.

This prospective longitudinal study showed that adverse PTSD symptom trajectories (i.e., chronic; delayed vs. resilient; recovery) during the first year following (suspected) serious injury were associated with higher levels of PTSD symptoms at 12–15 years. More exposure to additional potentially traumatic events and recent life stressors were associated with higher PTSD symptoms at 12–15 years post-trauma. Moreover, we found early post-trauma predictors (younger age at trauma; greater perceived impact of previous potentially traumatic events; and higher acute post-traumatic heart rate) with predictive value up to 12–15 years post-trauma. Interestingly, there was a high consistency between one-year PTSD and its previously observed early post-trauma predictors with long-term PTSD outcomes. This suggests that approaches related to early identification, and subsequent effective treatment, of individuals at risk for one-year PTSD may also hold more long-term beneficial effects for PTSD outcome.

## Supplementary information

Supplementary file A B C D

## Data Availability

The data of the study and the code to produce the results described in this paper are available at Open Science Framework (OSF; https://osf.io/myfu8/). The study is registered in the FAIR Traumatic Stress Data Sets library of the Global Collaboration on Traumatic Stress (GCTS).
