# Supplementary material for: PTSD course and predictors in a 15 year longitudinal cohort following suspected serious injury
Source: Npj Ment Health Res. 2025 Aug 7;4:35. doi: 10.1038/s44184-025-00153-7 (PMC12331889; doi:10.1038/s44184-025-00153-7)
Supplement: Supplementary file 1 — Supplementary file A B C D [file 44184_2025_153_MOESM1_ESM.docx]

**Supplementary file A: Sample Characteristics**

**Table A**

*Sample characteristics*

|  | Total (*N* = 155)  *n* (%) |
| --- | --- |
| Gender (women) | 57 (36.8%) |
| Age in years, *M* (*SD*) | 54.50 (12.6) |
| Netherlands as country of origin | 137 (88.4%) |
| Relationship status |  |
| Married/cohabitating/committed relationship | 136 (87.8%) |
| Divorced/widowed | 6 (3.9%) |
| No committed relationship | 12 (7.7%) |
| Children (yes) | 116 (74.8%) |
| Currently employed | 35 (22.6%) |
| Education, highest completed |  |
| Primary education/high school/secondary education | 24 (15.5%) |
| Secondary vocational education | 78 (50.3%) |
| Higher vocational education or University | 50 (32.3%) |
| Index trauma, type |  |
| Traffic accident | 92 (59.4%) |
| Physical violence | 2 (1.3%) |
| Work-related accident | 14 (9%) |
| Fall from height | 5 (9.7%) |
| Other | 4 (2.6%) |
| Injury (yes) | 148 (95.5%) |
| Severe injury based on Injury severity index (ISS) score of 16 or higher (yes) | 35 (22.6%) |
| Injury severity index (ISS), *M (SD*) | 9.99 (10.11) |
| Hospital admission (yes) | 97 (62.6%) |
| Intensive Care Unit (ICU) admission (yes) | 19 (12.3%) |
| Glasgow Coma Scale (GSC), *M (SD*) | 14.27 (2.36) |
| GSC Eye opening, *M (SD*) | 3.77 (0.72) |
| GSC Motor response, *M (SD*) | 5.78 (0.92) |
| GSC Verbal response, *M (SD*) | 4.61 (1.03) |
|  |  |

**Supplementary file B: Multiple Imputation**

**Table B**

*Overview of Missing Data*

|  | *n (%)* |
| --- | --- |
| Heart rate at ED admittance | 53 (34.2%) |
| Systolic blood pressure at ED admittance | 46 (29.7%) |
| Cortisol (nmol/L) at ED admittance | 77 (49.7%) |
| Thyroid stimulating hormone (mE/L) at ED admittance | 103 (66.5%) |
| Free thyroxine (pmol/L) at ED admittance | 102 (65.8%) |
| DHEAS (nmol/L) at ED admittance | 80 (51.6%) |
| Nonopioid aneasthics administration within 48 hours post-trauma (number of dosages) | 36 (23.2%) |
| Nonopiate analgesics administration within 48 hours post-trauma (number of dosages) | 36 (23.2%) |
| Opiate administration within 48 hours post-trauma (number of dosages) | 36 (23.2%) |
| Perceived life threat (yes) | 6 (3.9%) |
| Self-reported amnesia (yes) | 3 (1.9%) |
| ED admittance minutes from sunrise | 14 (9%) |
| Age at ED admittance (in years) | 0 (0%) |
| Prior traumatic events (number of types) | 0 (0%) |
| Total impact of prior traumatic events | 6 (4.5%) |

**Supplementary file C: Predictor Matrix Multiple Imputation**

**Table C**

*Predictor Matrix for Multiple Imputation*

|  | Free thyroxine (pmol/L) at ED admittance | Thyroid stimulating hormone (mE/L) at ED admittance | Cortisol (nmol/L) at ED admittance | Perceived life threat (yes) | DHEAS (nmol/L) at ED admittance | Systolic blood pressure at ED admittance | Total impact of prior traumatic events | Age at ED admittance (in years) | Non-opioid anesthetics administration within 48 hours post-trauma (number of dosage) | Self-reported amnesia (yes) | Non-opiate analgesics administration within 48 hours post-trauma (number of dosage) | Opiate administration within 48 hours post-trauma (number of dosage) | Prior traumatic events (number of types) | Heart rate at ED admittance | ED admittance in minutes since sunrise |
| --- | --- | --- | --- | --- | --- | --- | --- | --- | --- | --- | --- | --- | --- | --- | --- |
| CAPS-5 total score | 0 | 0 | 0 | 0 | 0 | 0 | 0 | 0 | 0 | 0 | 0 | 0 | 0 | 0 | 0 |
| Free thyroxine (pmol/L) at ED admittance | 0 | 0 | 1 | 0 | 1 | 0 | 0 | 0 | 0 | 0 | 0 | 0 | 0 | 0 | 0 |
| Thyroid stimulating hormone (mE/L) at ED admittance | 0 | 0 | 1 | 0 | 1 | 0 | 1 | 0 | 1 | 0 | 1 | 1 | 1 | 0 | 1 |
| Cortisol (nmol/L) at ED admittance | 0 | 0 | 0 | 0 | 0 | 0 | 0 | 0 | 0 | 0 | 0 | 0 | 0 | 0 | 0 |
| Perceived life threat (yes) | 0 | 0 | 0 | 0 | 0 | 0 | 0 | 0 | 0 | 0 | 0 | 0 | 0 | 0 | 0 |
| DHEAS (nmol/L) at ED admittance | 0 | 0 | 0 | 0 | 0 | 0 | 0 | 1 | 1 | 0 | 1 | 1 | 0 | 0 | 0 |
| Systolic blood pressure at ED admittance | 0 | 0 | 0 | 0 | 0 | 0 | 0 | 0 | 0 | 0 | 0 | 0 | 0 | 0 | 0 |
| Total impact of prior traumatic events | 0 | 1 | 0 | 0 | 0 | 0 | 0 | 0 | 0 | 0 | 0 | 0 | 1 | 0 | 0 |
| Age at ED admittance (in years) | 0 | 0 | 0 | 0 | 0 | 0 | 0 | 0 | 0 | 0 | 0 | 0 | 0 | 0 | 0 |
| Non-opioid anesthetics administration within 48 hours post-trauma (number of dosage) | 0 | 1 | 0 | 0 | 1 | 0 | 0 | 0 | 0 | 0 | 1 | 1 | 0 | 0 | 0 |
| Self-reported amnesia (yes) | 0 | 0 | 0 | 0 | 0 | 0 | 0 | 0 | 0 | 0 | 0 | 0 | 0 | 0 | 0 |
| Non-opiate analgesics administration within 48 hours post-trauma (number of dosage) | 0 | 1 | 0 | 0 | 1 | 0 | 0 | 0 | 1 | 0 | 0 | 1 | 0 | 0 | 0 |
| Opiate administration within 48 hours post-trauma (number of dosage) | 0 | 1 | 0 | 0 | 1 | 0 | 0 | 0 | 1 | 0 | 1 | 0 | 0 | 0 | 0 |
| Prior traumatic events (number of types) | 0 | 0 | 0 | 0 | 0 | 0 | 0 | 0 | 0 | 0 | 0 | 0 | 0 | 0 | 0 |
| Heart rate at ED admittance | 0 | 0 | 0 | 0 | 0 | 1 | 0 | 0 | 0 | 0 | 0 | 0 | 0 | 0 | 0 |
| ED admittance in minutes since sunrise | 0 | 1 | 0 | 0 | 0 | 0 | 0 | 0 | 0 | 0 | 0 | 0 | 0 | 0 | 0 |
| Another person died during traumatic event | 0 | 0 | 0 | 0 | 0 | 0 | 0 | 0 | 0 | 0 | 0 | 0 | 0 | 0 | 1 |
| Acquaintance died during traumatic event | 0 | 0 | 0 | 0 | 0 | 0 | 0 | 0 | 0 | 0 | 0 | 0 | 0 | 0 | 1 |
| Antihistaminic administration within 48 hours post-trauma | 0 | 0 | 0 | 0 | 0 | 0 | 0 | 0 | 0 | 1 | 0 | 0 | 0 | 0 | 0 |
| Anticoagulation administration within 48 hours post-trauma | 0 | 0 | 0 | 0 | 0 | 0 | 0 | 0 | 0 | 0 | 0 | 0 | 0 | 1 | 0 |
| Against hypertension administration within 48 hours post-trauma | 0 | 1 | 0 | 0 | 0 | 0 | 0 | 0 | 1 | 0 | 1 | 1 | 0 | 0 | 0 |
| Hospital admission | 1 | 0 | 1 | 0 | 0 | 0 | 0 | 0 | 0 | 0 | 0 | 0 | 0 | 0 | 0 |
| ICU admission | 0 | 1 | 0 | 0 | 0 | 0 | 0 | 0 | 1 | 0 | 1 | 1 | 0 | 0 | 0 |
| Time in hospital | 0 | 0 | 0 | 0 | 0 | 0 | 0 | 0 | 0 | 0 | 0 | 0 | 0 | 1 | 0 |
| Education | 1 | 0 | 0 | 0 | 0 | 0 | 0 | 0 | 0 | 0 | 0 | 0 | 0 | 0 | 0 |
| Work hours | 1 | 0 | 0 | 0 | 0 | 0 | 0 | 0 | 0 | 0 | 0 | 0 | 0 | 0 | 0 |
| ISS categories | 0 | 0 | 0 | 0 | 1 | 0 | 0 | 0 | 0 | 0 | 0 | 0 | 0 | 0 | 0 |
| Anxious | 0 | 0 | 1 | 0 | 0 | 0 | 0 | 0 | 0 | 1 | 0 | 0 | 0 | 1 | 0 |
| Furious | 0 | 0 | 0 | 1 | 0 | 0 | 0 | 0 | 0 | 0 | 0 | 0 | 0 | 0 | 0 |
| Helpless | 0 | 0 | 0 | 0 | 0 | 0 | 0 | 0 | 0 | 0 | 0 | 0 | 1 | 0 | 0 |
| Disgust | 0 | 0 | 1 | 0 | 0 | 0 | 1 | 0 | 0 | 0 | 0 | 0 | 1 | 0 | 0 |
| Sadness | 0 | 0 | 0 | 1 | 0 | 0 | 0 | 0 | 0 | 0 | 0 | 0 | 0 | 0 | 0 |
| Emotional total score | 0 | 0 | 0 | 0 | 0 | 0 | 1 | 0 | 0 | 0 | 0 | 0 | 0 | 0 | 0 |
| Tired item 1 | 0 | 0 | 0 | 1 | 0 | 0 | 1 | 0 | 0 | 0 | 0 | 0 | 1 | 0 | 0 |
| Tired item 2 | 1 | 1 | 1 | 0 | 0 | 1 | 0 | 0 | 0 | 0 | 0 | 0 | 0 | 0 | 0 |
| Tired item 3 | 1 | 0 | 1 | 0 | 0 | 1 | 0 | 0 | 0 | 0 | 0 | 0 | 0 | 0 | 0 |
| Tired item 4 | 1 | 0 | 1 | 0 | 0 | 0 | 0 | 0 | 0 | 0 | 0 | 0 | 0 | 0 | 0 |
| PDI item 1 | 1 | 1 | 1 | 0 | 0 | 0 | 0 | 0 | 0 | 0 | 0 | 0 | 0 | 0 | 0 |
| PDI item 4 | 0 | 0 | 0 | 0 | 1 | 0 | 0 | 1 | 0 | 0 | 0 | 0 | 0 | 0 | 0 |
| PDI item 6 | 0 | 0 | 0 | 0 | 0 | 0 | 0 | 0 | 0 | 0 | 0 | 0 | 0 | 0 | 0 |
| PDI item 8 | 0 | 1 | 0 | 0 | 0 | 0 | 0 | 0 | 0 | 0 | 0 | 0 | 0 | 0 | 0 |

*Note. Included = 1, not included = 0.*

**Supplementary file D: Correlation Matrix**

**Table D**

*Pearson Correlations between included 15 predictors*

|  | Heart rate | Systollic blood pressure | Cortisol | TSH | FT | DHEAS | Nonopioid aneasthics | Nonopiate analgesics | Opiate | Perceived life threat | Self-reported amnesia | Time at ED | Age at ED | Prior traumatic events | Impact of prior traumatic events |
| --- | --- | --- | --- | --- | --- | --- | --- | --- | --- | --- | --- | --- | --- | --- | --- |
|  |  |  |  |  |  |  |  |  |  |  |  |  |  |  |  |
|  |  |  |  |  |  |  |  |  |  |  |  |  |  |  |  |
| Heart rate | 1.00 | .03 | .03 | .02 | .09 | .05 | -.03 | -.03 | -.12 | .11 | -.16 | .25* | .01 | .05 | .16 |
| Systollic blood pressure |  | 1.00 | -.06 | -.12 | -.06 | .09 | -.09 | -.09 | -.15 | .08 | .10 | .14 | .12 | .07 | .11 |
| Cortisol |  |  | 1.00 | .08 | -.06 | .15 | .22 | .22 | .23 | -.09 | -.09 | -.27* | -.06 | -.19 | -.00 |
| TSH |  |  |  | 1.00 | -.11 | .08 | .46** | .46** | .45** | -.09 | .01 | .36* | -.06 | -.34* | -.36** |
| FT |  |  |  |  | 1.00 | .19 | .08 | .08 | .20 | -.19 | -.01 | -.06 | .01 | -.03 | -.11 |
| DHEAS |  |  |  |  |  | 1.00 | .32** | .32** | .41** | -.01 | .04 | .06 | -.39** | .02 | .01 |
| Nonopioid aneasthics |  |  |  |  |  |  | 1.00 | .99** | .70** | .04 | -.10 | -.01 | -.05 | -.17 | .04 |
| Nonopiate analgesics |  |  |  |  |  |  |  | 1.00 | .71** | .04 | -.10 | -.03 | -.05 | -.17 | .08 |
| Opiate |  |  |  |  |  |  |  |  | 1.00 | .07 | -.07 | -.12 | -.05 | -.20* | -.17 |
| Perceived life threat |  |  |  |  |  |  |  |  |  | 1.00 | .24** | .04 | .05 | .17* | .18* |
| Self-reported amnesia |  |  |  |  |  |  |  |  |  |  | 1.00 | -.01 | .07 | .25** | .19* |
| ED admittance minutres from sunrise |  |  |  |  |  |  |  |  |  |  |  | 1.00 | -.05 | .01 | -.04 |
| Age at ED admittance |  |  |  |  |  |  |  |  |  |  |  |  | 1.00 | .26** | .12 |
| Prior traumatic events |  |  |  |  |  |  |  |  |  |  |  |  |  | 1.00 | .77* |
| Impact of prior traumatic events |  |  |  |  |  |  |  |  |  |  |  |  |  |  | 1.00 |

*Note. * = Correlation is significant at the p/alpha 0.05 level (2-tailed).
** = Correlation is significant at the 0.01 level (2-tailed).*
